# Unusual sized cecal mass presenting without obstruction: a case report

**DOI:** 10.1186/1757-1626-2-131

**Published:** 2009-02-06

**Authors:** Ali R Elyassi, Kevin Lin-Hurtubise, Ronald Gagliano

**Affiliations:** 1 Postgraduate Resident Year2, Department of Oral and Maxillofacial Surgery, Tripler Army Medical Center, Honolulu, HI, USA; 2 Professor and Surgical Oncologist, Department of Surgery, Tripler Army Medical Center, Honolulu, HI, USA; 3 Program Chief, Department of Surgery, Tripler Army Medical Center, Honolulu, HI, USA

## Abstract

**Background:**

Colorectal cancer (CRC) is a common and lethal disease with thousands of new cases of large bowel cancer diagnosed in the United States each year. Colorectal cancer frequently causes obstruction of the large bowel. Cases of obstructions and perforations have been documented with masses well below 6 cm in diameter. Obstruction and/or perforation are important predictors of prognosis with respect to colorectal carcinomas.

**Case Presentation:**

The following is the presentation of an unusual case of a 13 × 12 × 16 cm colonic adenocarcinoma without any signs of obstruction or perforation.

**Conclusion:**

Perioperative mortality rates for colorectal cancer with obstruction have been documented anywhere from 5% to 47.6%, making obstruction an important predictive factor in long-term patient survival. Although a correlation between obstruction and patient outcome has been determined, a correlation between colorectal tumor size/staging and obstruction has yet to be investigated.

## Introduction

Colorectal cancer (CRC) is a common and lethal disease with over 150,000 new cases of large bowel cancer diagnosed each year in the United States[1]. In the U.S., obstruction of the large bowel is most frequently caused by colorectal cancer[2]. Bowel obstruction occurs when the normal flow of bowel contents is interrupted. Small bowel obstruction in patients with cancer is a common and difficult problem and has been described in up to 28 percent of patients with colorectal carcinoma[3]. In a study by Maurer, CA et al, of 69 patients with large bowel obstruction, 38 percent were associated with colorectal carcinoma[4]. Cases of obstructions and perforations have been documented with masses well below 6 cm in diameter. The following is the presentation of an unusual case of a 13 × 12 × 16 cm colonic adenocarcinoma without any signs of obstruction or perforation.

## Case Presentation

A 58 year old Caucasian male with a history of iron deficiency anemia and severe depression presented with weakness and shortness of breath, along with a loss of appetite and thirty pound weight loss. He also has a long history of psychological issues, including extensive illicit drug use, bipolar personality disorder, anxiety, chronic depression, and suicidal tendencies. He denied any constipation, diarrhea, tenesmus, melena, hematochezia, nausea, vomiting, or abdominal pain. He did, however, report feeling a firm mass in his right upper and lower abdominal quadrants, which he claims has grown in size over the years.

Upon physical examination, the patient was found to appear thin, pale, but comfortable. He was found to have normal bowel sounds in all four quadrants; however, there was a large firm, non-tender mass palpated in the right upper and lower quadrants. Also, bilateral mildly reducible inguinal hernias and a large umbilical hernia were detected. Patient's exam was otherwise normal.

**Figure 1:**
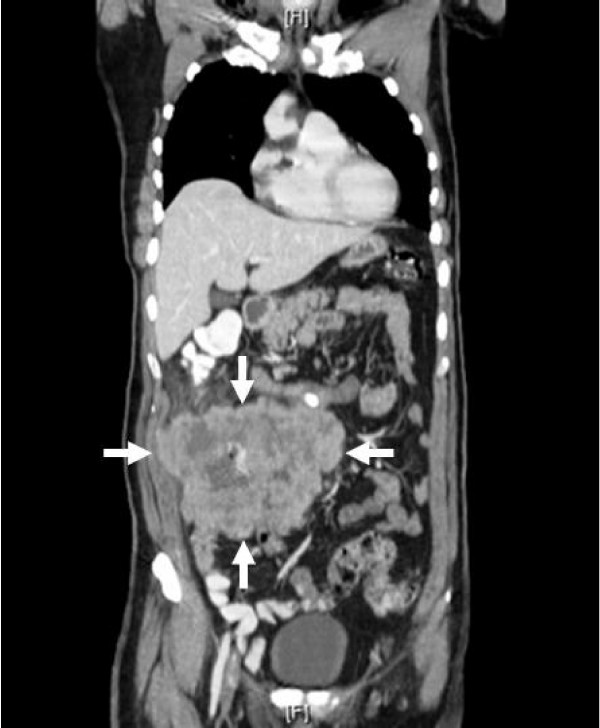
**Frontal view of Computed Tomography (CT), which revealed a large 13 × 12 × 16 cm right-sided ill-defined soft tissue mass involving the ascending colon**.

**Figure 2:**
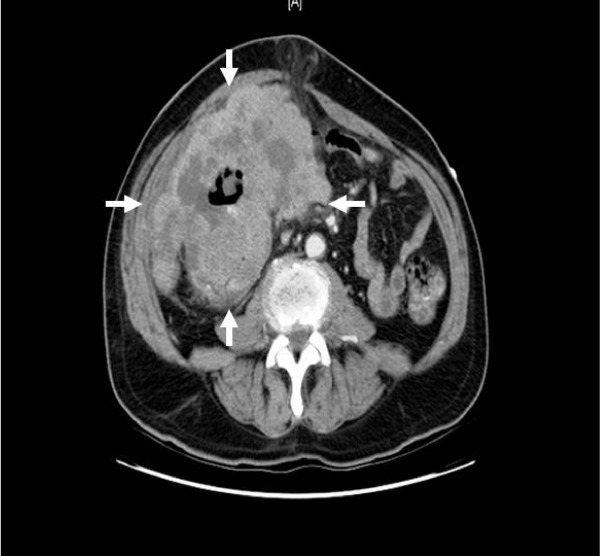
**Axial view of Computed Tomography (CT), which revealed 13 × 12 × 16 cm right-sided ill-defined soft tissue mass involving the ascending colon**.

The patient presented with a hemoglobin/hematocrit ratio of 7/25.7 and an albumin level of 2.9. He was therefore admitted for blood transfusions and further workup of a mass detected in his upper and lower right abdominal quadrants. Computed Tomography (CT) with contrast was performed which showed a large 13 × 12 × 16 cm right-sided ill-defined soft tissue mass involving the ascending colon (Fig 1 and 2). CT-guided fine needle aspiration and core biopsy were also performed which revealed adenocarcinoma. Carcinoembryonic antigen (CEA) was 69.7.

**Figure 3:**
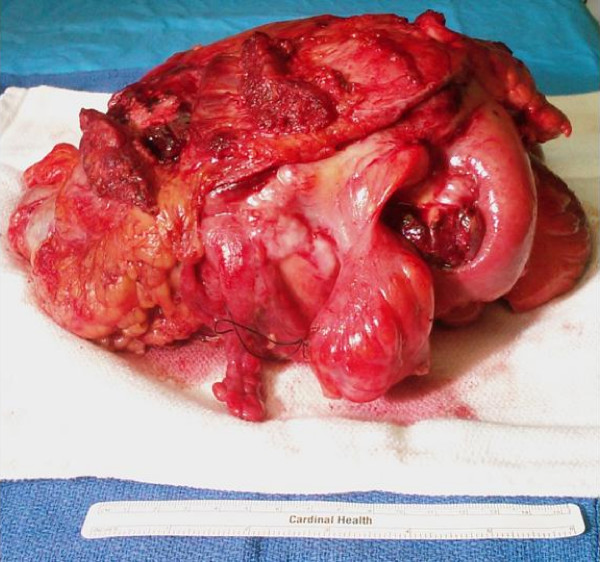
**Colonic mass which was surgically resected en bloc**.

After a week of total parenteral nutrition, the patient was taken to the operating room to undergo tumor resection under general anesthesia. Adequate exposure was attained by a vertical incision across the midline of the abdomen with a transverse extension to the right. Surgical planes were created to resect the colonic mass en bloc with the invaded appendix, ascending/transverse colon, loop of ileum, and right abdominal wall/retroperitoneum. Liver, ileocolic mesentery, and para-aortic regions were without adenopathy or mass. Proximal and distal surgical margins were clear and no residual tumor was left behind. Pathology reported the greater than 6 lb mass (Fig 3) to have 0/16 nodes positive for metastatic adenocarcinoma.

The patient has recovered well without complications, except for mild ileus, and was discharged on postoperative day fourteen to follow up for possible adjuvant chemotherapy.

## Discussion

Although the patient underwent surgery without complications, there is no doubt that his mental illness prevented him from seeking medical care sooner. It is unusual that the abdominal mass grew as large as it did without signs of bowel obstruction or perforation.

Small and/or large bowel obstruction in patients with cancer is a common and difficult problem and has been described in 28–38 percent of patients with colorectal carcinoma[3,4]. Cases of obstructions and perforations have been documented with masses well below 6 cm in diameter. This is an unusual case of a 13 × 12 × 16 cm colonic adenocarcinoma without any signs of obstruction or perforation.

Obstruction and/or perforation are important predictors of prognosis with respect to colorectal carcinomas. In adenocarcinoma of the colon, obstruction and perforation have been documented to have occurred at the site of the neoplasm and/or proximal. In one study alone, close to 2000 cases of colorectal adenocarcinoma with obstruction and perforation were examined and showed that both events carry a poor prognosis[5]. In a study by Wang et al, of 256 patient outcomes examined, survival rates were significantly lower in obstructed patients. This study also found obstruction status as an independent prognostic factor for patients with right colon carcinoma[6].

Studies across the world consistently show poorer outcomes for patients with obstructive colorectal cancer as opposed to non-obstructed cancer. A study performed in Asia documented the perioperative mortality rate for colorectal cancer with obstruction to be 5% and the 5-year survival to be 33%[5]. Zucchetti et al showed that radically resected obstructed patients had a disease-related death rate of 47.6% versus 16.3% for non-obstructed ones. Their study, performed in Europe, concluded that obstruction itself represented an independent unfavorable factor negatively affecting long term prognosis, even after radical resection.[7] In another study done in South America, Torres-Zavala et al showed survival rate of patients with obstructive colorectal cancer to be 9.5% versus 35.1% for non-obstructive ones[8].

The cancerous mass removed from the patient in this case was Stage IIB (T4, N0, M0), according to the American Joint Committee on Cancer staging system for colorectal cancer (Table 1)[9].

**Table 1:** TNM Staging System for Colorectal Cancer

Primary Tumor (T)
Tis: Carcinoma in situ; intraepithelial (within glandular basement membrane) or invasion of lamina propria (intramucosal)

T1: Tumor invades submucosa

T2: Tumor invades muscularis propria

T3: Tumor invades through the muscularis propria into the subserosa, or into nonperiotonealized pericolic or perirectal tissues

T4: Tumor directly invades other organs or structures, and/or perforates visceral peritoneum

**Regional Lymph Node (N)**

NX: Regional nodes cannot be assessed

N0: No regional node metastases

N1: Metastasis in 1 to 3 regional lymph nodes

N2: Metastasis in 4 or more regional lymph nodes

**Distant Metastasis (M)**

MX: Distant metastasis cannot be assessed

M0: No distant metastasis

M1: Distant metastasis

**Stage Groupings**

Stage 0: T_is_, N0, M0

Stage I: T1-2, N0, M0

Stage IIA: T3, N0, M0

Stage IIB: T4, N0, M0

Stage IIIA: T1-2, N1, M0

Stage IIIB: T3-4, N1, M0

Stage IIIC: Any T, N2, M0

Stage IV: Any T, Any N, M1


The original source for this material is the AJCC Cancer Staging Manual, Sixth Edition (2002) published by Springer-Verlag New York, Inc[6].

In this case, the tumor had invaded the retroperitoneal tissue (T4), zero out of sixteen nodes were found positive (N0), and no distant metastasis was discovered (M0). It is highly unusual that this Stage IIB (T4, N0, M0) tumor was found without any signs of obstruction or perforation.

Nevertheless, one cannot dismiss the fact that the patient's mental status could have "blunted" his true physical presentation. In other words, with chronically depressed individuals, such as in this case, it is difficult to determine whether the loss of appetite and weight loss is secondary to their mental illness(es) or cancer. Even with a thorough history and physical, the clinician can be misguided, especially if the patient is not presenting other symptoms that would make him/her more suspicious of other underlying conditions.

## Conclusion

The incidence of colorectal cancer makes it a very common disease process across the world. Perioperative mortality rates for colorectal cancer with obstruction have been documented anywhere from 5% to 47.6%, making obstruction an important predictive factor in long-term patient survival. Although a correlation between obstruction and patient outcome has been determined, a correlation between colorectal tumor size/staging and obstruction has yet to be investigated.

## Consent

Written and verbal informed consent was obtained from the patient for publication of this case report and accompanying images. A copy of the written consent is available for review by the Editor-in-Chief of this journal.

## Competing interests

The views expressed in the submitted abstract/manuscript are those of the author(s) and do not reflect the official policy or position of the Department of the Army, Department of Defense, or the U.S. Government. Also, the authors undersigned disclose that there are no financial, economic, commercial, and/or professional interests related to the topics presented in the manuscript. The authors declare that they have no competing interests.

## Authors' contributions

AE and KL were both involved in direct patient care, the surgery, perioperative management, literature review, and writing the manuscript. RG was a contributor in writing the manuscript. All authors helped read, edit, and approve the final manuscript.
